# Membrane-Integrated Organ-on-a-Chip Systems

**DOI:** 10.3390/bios16100534

**Published:** 2026-09-24

**Authors:** Moldir Kali, Almas Rakhymzhanov, Gulsim Kulsharova

**Affiliations:** 1 Department of Electrical and Computer Engineering, School of Engineering, Nazarbayev University, Astana 010000, Kazakhstan; 2 Nanofabrication Core Lab, King Abdullah University of Science and Technology (KAUST), Thuwal 23955-6900, Saudi Arabia

**Keywords:** microfluidics, organ-on-a-chip, organ-on-a-chip devices, microphysiological systems, membrane-integrated organs-on-chip, membrane fabrication, basement membrane

## Abstract

Membranes play a key role in many organ-on-a-chip (OOC) platforms due to nutrient and mass transport, barrier formation, and mechanical signaling. However, despite their key role in OOC devices, the relationship between membrane design parameters and device performance is limited in the literature. Here, this review gives a critical overview of not only the types of membranes for OOC devices (natural, synthetic, hybrid, and emerging functional membranes) but also defines the key engineering design requirements for the selection and integration of membranes with microfluidic OOC platforms. Additionally, it presents types of organ-specific OOC devices (lung, kidney, liver, blood–brain barrier, and intestine models), highlighting existing challenges and limitations in the fabrication of such platforms. The paper also presents future directions toward smart, multifunctional membranes.

## 1. Introduction

Membrane-integrated organ-on-a-chip (OOC) platforms are largely dependent on the engineered barrier separating cell or tissue compartments. In many OOC devices, this barrier is emulated by a porous membrane that plays such roles as regulating mass transport, mechanical coupling, and structural stability for cell culture. The membrane controls molecular exchange, cell–cell communication, and mechanical stimuli, while defining the physiological microenvironment experienced by cells [1].

As OOC technologies have expanded to model the heart, gut, liver, kidney, brain, and other organs, membranes are required to support increasingly complex biological functions, including shear transmission, nutrient and gas exchange, and optical accessibility for real-time imaging [2,3]. Meeting these demands introduces conflicting engineering requirements: membranes must remain mechanically compliant while maintaining strength, chemical stability under flow, and biologically relevant surface properties [4,5].

Unlike previous reviews that primarily describe membrane materials or organ-specific OOC platforms, this review organizes the field around engineering design constraints, linking membrane material properties, microstructure, and fabrication strategies to device-level performance. We therefore propose a design-constraint framework for membrane-integrated OOC systems that identifies the key parameters governing device functionality, including material composition, mechanical suitability, transport characteristics, and membrane integration. Specifically, this framework organizes membrane design decisions into four design-constraint domains, which are systematically discussed in Section 2: (i) material composition and biocompatibility, which determine biological characteristics and the possibility of regulatory considerations and manufacturability; (ii) mechanical properties assessed separately at the material level (intrinsic modulus, viscoelasticity) and at the device level (effective compliance taking into account thickness, geometry, and clamping/bonding); (iii) mass transport, encompassing pore size, porosity, pore density, interconnectivity, tortuosity, and solute-specific permeability; and (iv) fabrication and integration compatibility, covering achievable feature sizes, bonding strategy, and long-term interface stability. This framework is applied in two stages: Section 2 discusses different membrane classes with respect to these domains, while Section 3 applies the framework to organ-specific membrane-integrated OOC platforms and summarizes key membrane and device characteristics in Table 1, enabling systematic comparison across membrane types and organ models.

Several previous reviews have focused on membranes in OOC platforms, but each has a narrower or different framework than the present work. Corral-Najera et al. [5] and Youn and Kim [34] catalog membrane materials, their physical/biochemical properties, and fabrication methods for membrane selection, but do not organize these materials within a device-level engineering framework or compare organ systems using a common set of parameters. Similarly, Rahimnejad et al. [35] review engineered biomimetic membranes organized by target organ (liver, kidney, intestine, lung, skin, heart, vasculature, and brain), comparing membrane preparation and characterization against native extracellular matrix (ECM) features for each system; while comprehensive in scope, this organization does not extract transferable, cross-organ design principles or distinguish material- from device-level membrane properties, which is the central aim of the present framework. Salimbeigi et al. [36] consider the physical and mechanical properties of native basement membranes and strategies for replicating them but focus on the biological target (the basement membrane itself) rather than on device-level integration constraints such as adhesion, fabrication compatibility, or effective compliance. Organ-specific reviews, such as those of Zhang et al. [37] (lung, organized around disease pathophysiology) and Nguyen et al. [38] (kidney, a systematic review of kidney chip models in general), provide depth within a single organ system but consider membranes as only one of several design elements, preventing comparisons across organs. This review differs from the previously mentioned works in three respects: (1) it uses the limitations of membrane engineering as an organizing principle, rather than membrane chemistry, bone marrow biology, or organ identity; (2) it explicitly distinguishes between material-level properties (e.g., bulk Young’s modulus, surface chemistry) and device-level properties (e.g., effective membrane compliance, integrated permeability), a distinction that is often conflated in the literature; and (3) it summarizes design trade-offs for five organ systems using a common set of parameters (Table 1), allowing the identification of transferable design principles rather than organ-specific observations.

In this paper, membrane types are introduced; then, the engineering design constraints governing their integration are outlined, and representative organ-specific OOC platforms are reviewed. Figure 1 provides a schematic overview of membrane-integrated microfluidic platforms, highlighting the role of membranes as functional interfaces linking engineering design to biological function and organ-specific applications.

### Literature Search Methodology

A literature search was conducted using Scopus with keywords “nanofiber membrane”, “organ-on-a-chip”, “membrane integration”, “basement membrane”, “natural membranes”, “hybrid membranes”, “nanofiber membrane fabrication”, “lung-on-a-chip”, “kidney-on-a-chip”, “gut-on-a-chip”, “liver-on-a-chip”, and “BBB chip,” covering publications from 2010 to 2025. The search was conducted in June 2026. Boolean combinations (e.g., “membrane” and “organ-on-a-chip” and organ-specific term) were used to refine the search.

Studies were included if they (i) were peer-reviewed, English-language, primary research or review articles published between 2010 and 2025; (ii) described a microfluidic OOC device incorporating a physical membrane component; and (iii) reported at least one membrane design or performance parameter (material composition, thickness, pore size/porosity, permeability, or mechanical property). Conference abstracts without an accessible full text, patents, and studies describing membrane-free OOC architectures without a comparative membrane-based counterpart were excluded unless cited for contrast. Screening titles and full texts against the criteria yielded 78 references synthesized across Section 2, Section 3, and Section 4.

## 2. Membranes for Organ-on-a-Chip Systems

Native basement membranes (BMs) that organ-on-a-chip (OOC) platforms aim to replicate vary between organs. However, in general, in nature, they are made of natural materials with characteristic fibrous topology, thickness, roughness, porosity, and other structural properties.

Membrane properties are discussed at two distinct levels. Material-level properties describe the free-standing film—composition, intrinsic Young’s modulus, viscoelasticity, surface chemistry, and as-fabricated pore size/porosity—typically measured by bulge testing or AFM [2]. Device-level properties describe the same membrane once clamped and bonded within the chip—effective compliance, integrated permeability, and interface stability—which can diverge substantially from material-level values due to boundary constraints and bonding-induced pre-stress [4]. This distinction is applied throughout Section 2.

Membranes used for many OOC designs, depending on their composition and origin, can be classified into synthetic, hybrid, or composite types, each offering distinct advantages and limitations in replicating physiological conditions. Overall, the diversity of membrane materials and fabrication strategies reflects the lack of a universal solution capable of fully recapitulating native BM properties. Each membrane class addresses specific aspects of physiological relevance, such as mechanical support, selective permeability, or biochemical signaling, while simultaneously introducing inherent limitations. As a result, membrane selection in OOC platforms remains highly application-dependent and requires careful balancing of biological fidelity, mechanical robustness, and manufacturing feasibility. Membranes are classified below primarily by type (natural, synthetic, hybrid); fabrication route (e.g., in situ generation) and functional behavior (e.g., stimuli-responsiveness) are cross-cutting attributes that apply within, rather than alongside, this classification and are noted accordingly.

*Natural (biological) membranes.* Natural membranes are derived from biological sources such as proteins and polysaccharides. Because of their biocompatibility, biodegradability, and environmentally friendly properties, they offer close biochemical similarity to native BMs and are widely used to fabricate biomimetic BMs, although this biological fidelity comes with well-documented trade-offs in batch-to-batch reproducibility and mechanical robustness that are discussed further below. Such materials include collagen, laminin, fibrin, decellularized extracellular matrix (dECM), gelatin, alginate (ALG), and hyaluronic acid (HA) [39]. These natural polymers have been extensively used to create hydrogels, electrospun scaffolds, or coatings to provide synthetic materials with the desired biological properties [40].

Recent advances in natural membranes focus on enhancing their mechanical stability, degradation control, and organ-specific functionality to reproduce physiological barriers in OOC systems. For instance, scientists developed collagen I-based enzymatically degradable membranes with tunable stiffness and transparency, demonstrating that membrane degradation can be controlled by adjusting the collagen concentration and crosslinking density to mimic in vivo BMs [41]. Similarly, the authors reported that collagen membranes maintain optical transparency and elasticity compatible with cyclic stretching in lung-on-chip (LOC) devices, enabling stable epithelial–endothelial co-cultures [2]. In addition, sac-like and stretchable hydrogel membranes fabricated via micromolding or 3D printing enable the replication of curved and dynamic interfaces such as alveolar and vascular barriers [42]. However, these favorable properties are typically demonstrated over short culture windows; hydrogel membranes remain susceptible to swelling, hydrolytic or enzymatic degradation, and dimensional instability over multi-day to multi-week perfusion, which can compromise barrier integrity and is discussed further below. These developments collectively advance the physiological relevance of OOC platforms by allowing membranes to participate actively in cell signaling, remodeling, and mechanical feedback.

*Synthetic membranes.* These membranes typically consist of synthetic materials such as polydimethylsiloxane (PDMS), polycarbonate (PC), polyethylene terephthalate (PET), or polytetrafluoroethylene (PTFE) [34]. Such a type of synthetic membrane possesses good mechanical characteristics that enable easy manipulation, easy incorporation into a microfluidic chip, and stable cell support. They are also commercially viable since they can be scaled up, although their extensive availability, as well as mechanical capacities and their material properties, are significantly disparate from physiological organ functions [43].

Recent developments aim to improve the physiological relevance of synthetic membranes. For instance, micromolded porous PDMS membranes have been optimized for gut-on-chip applications, offering better reproducibility and integration [44]. Nevertheless, PDMS continues to experience issues with hydrophobicity and low-molecule absorption, which makes it necessary to use other polymers [45]. This adsorption behavior has particular implications for the pharmacological applications of OOC platforms. PDMS-based membranes are known to non-specifically adsorb small hydrophobic molecules and some proteins due to their hydrophobicity and porous bulk structure, which can artificially lower the apparent concentration of a drug candidate reaching the cell layer and bias dose–response measurements in drug screening studies [45]. This is particularly relevant for lipophilic compounds, where a significant fraction of the nominal dose may penetrate the PDMS bulk rather than reach the target cells, potentially leading to the underestimation of drug efficacy or toxicity. Given that many of the OOC platforms reviewed here (Section 3) are used solely for drug toxicity and permeability screening, this limitation is an important practical consideration when interpreting pharmacological data obtained with PDMS-based devices. Alternative polymers such as thermoplastic polyurethane (TPU) and cyclic olefin copolymer (COC) have emerged as promising PDMS substitutes, offering improved elasticity, optical transparency, and reduced drug absorption [46]. These innovations are proof of development in synthetic membranes, which are more similar to natural tissue mechanics and barrier functions. Although recent material innovations have improved synthetic membrane performance, balancing mechanical tunability, permeability control, and biological functionality remains challenging. These considerations have motivated the development of alternative membrane strategies that integrate synthetic robustness with biologically active components.

*Hybrid membranes.* Hybrid membranes are mixtures formed by the artificial combination of natural and synthetic materials. Natural and synthetic polymers have their own limitations, yet their combination takes into consideration their benefits and counters their limitations to create materials with physical and biochemical properties that are highly similar to those of natural BMs. Recent studies have demonstrated several types of hybridization strategies. One of the most common is material-based hybridization. The case of collagen/gelatin inserts in polycaprolactone (PCL) or PDMS has been shown to increase the biocompatibility and permeability of the system without affecting the mechanical stability of the dynamic culture system [5]. A recent example demonstrated a hybrid PCL–collagen electrospun membrane (~10 µm thick) integrated into a microfluidic LOC device, showing excellent biocompatibility to mimic basement membrane function. Hybrid membranes also offer improved adaptability to dynamic microfluidic environments, as the synthetic backbone provides resistance to deformation and rupture, while the natural component supports cell adhesion, signaling, and matrix remodeling.

Another emerging direction is method-based hybridization, which combines fabrication techniques such as electrospinning and 3D bioprinting, or phase separation and lithography, to construct multilayer or graded membranes that better reproduce tissue heterogeneity. For example, a sac-like hydrogel membrane fabricated via a template-free “soap bubble” method enabled a curved alveolar–capillary barrier in an LOC [42]. Similarly, combined fabrication approaches such as phase separation with micromolding have been reported to produce membranes with dual-scale porosity and surface patterning, enhancing control over transport and cell–matrix interactions [47]. Overall, both material-based and method-based hybridization strategies contribute to the generation of membranes with hierarchical architectures and multifunctional properties.

*In situ-generated fabrication* (a cross-cutting route applicable to natural or hybrid membranes). In situ-generated membranes offer an alternative fabrication strategy in which the membrane forms directly within the microfluidic device, without separate membrane manufacturing and integration steps. This method can facilitate device fabrication and minimize the probability of membrane damage during handling. Early work by researchers demonstrated that freestanding, semi-permeable chitosan membranes could be generated inside microfluidic channels through pH gradient-driven biofabrication, which can serve as a framework to grow membranes on chips [48]. Subsequently, in situ-generated extracellular matrix (ECM) has been used as a membrane-free intestinal tubule that can function as a barrier and polarize within a perfused microfluidic device [49]. Further, this concept was advanced by describing microfluidic gel patterning using a temporary membrane, by means of which hydrogel compartments can be fabricated in OOC models [50].

*Emerging functional attributes* (nanofibrous, decellularized ECM (dECM), stimuli-responsive). These are functional attributes rather than a distinct material class, and can be combined with the natural, synthetic, or hybrid membranes described above. Emerging functional membrane classes have been developed to replicate not only structural features but also the dynamic behavior of human tissues in OOC platforms, thus enhancing physiological relevance and enabling real-time responses to mechanical, chemical, and biochemical cues. OOC devices increasingly rely on advanced biomimetic membranes to mimic in vivo microenvironments, including tissue interfaces, fluidic shear stress, and cellular differentiation patterns [51]. A defining feature of these emerging membrane classes is their ability to actively participate in cell–matrix interactions rather than serving as passive structural supports.

Nanofibrous membranes with ECM-like architecture offer high surface-to-volume ratios, porous microstructures, and topographical cues that promote cell adhesion, migration, and differentiation within OOC systems [51]. dECM membranes provide complex biochemical cues and native proteins that guide cell behavior and organ-specific functions, enabling more accurate recapitulation of tissue microenvironments within chips [52]. A recurring limitation of dECM-based membranes, however, is incomplete control over composition: decellularization protocols and source tissue variability lead to substantial batch-to-batch differences in residual biochemical content, limiting reproducibility relative to defined synthetic materials [53]. Integration of controlled ECM matrices into OOC systems has been highlighted as a key strategy to improve the mimicry of native ECM properties and to overcome the limitations of purely synthetic membranes [53]. Patterned hydrogel membranes with controlled micro- and nano-topographies influence the mechanosensing and spatial organization of cells, important for replicating anisotropic tissue structure and physiological interfaces such as epithelial barriers.

Stimuli-responsive or “smart” membranes adapt their properties (e.g., stiffness, permeability, swelling behavior) to external cues such as pH, temperature, or mechanical forces, offering dynamic and responsive control over microenvironmental conditions. Smart hydrogel systems are increasingly incorporated into microphysiological platforms to enable the controlled release of biochemical signals and to mimic adaptive tissue behavior in response to changing physiological stimuli [51]. Hydrogels, made from native ECM materials, like Matrigel, collagen, or fibrin, can provide an intrinsically biocompatible solution with high water content and a good match to the mechanical compliance of soft tissues, while their lack of mechanical strength under perfusion may hinder their applications—in particular, hydrogel membranes are prone to dimensional changes due to swelling, hydrolytic or enzymatic degradation over multi-day/multi-week culture periods, and direct mechanical failure (rupture or delamination) during continuous perfusion. All of these phenomena should be assessed over the intended culture period, rather than at a single point in time [54]. Ultrathin polymer nanofilms, such as those below 1 µm in thickness on the order of silicon nitride, can effectively reduce the physical distance between cell layers while maintaining better similarity to native BMs than that of etched porous PC filters and thus better paracrine signaling between layers [55]. Collectively, these membrane classes—nanofibrous, dECM, patterned hydrogel, and stimuli-responsive membranes—provide strategies to emulate dynamic tissue behavior in OOC devices.

Overall, no single class of membranes dominates across all criteria. Natural membranes provide the closest biological fidelity but lack reproducibility and mechanical strength; synthetic membranes provide strength and scalability, but poorly mimic biomimetics; hybrid membranes partially fill this gap mechanically but still cannot reproduce the thickness of the natural basement membrane or nonlinear strengthening [36]; and in situ-engineered membranes avoid damage during handling but sacrifice control over thickness and porosity [48,49,50].

### 2.1. Membrane Necessity and Membrane-Free Alternatives

A physical membrane is not an obligatory feature of OOC design, but one solution to the broader requirement of controlled compartmentalization between cell populations. Whether a membrane is needed depends on the specific function required: a discrete, addressable barrier for transepithelial electrical resistance (TEER) measurement, a substrate for mechanical actuation, or independently tunable thickness and porosity all favor a membrane-based design. Conversely, interfaces where transport occurs primarily through an ECM-like hydrogel in vivo can instead be reconstructed as membrane-free architectures, in which cell-laden hydrogels are geometrically confined by surface tension pinning (e.g., phaseguide structures), laminar flow patterning, or biofabricated ECM barriers [49,52]. Membrane-free designs can better recapitulate the continuous, three-dimensional ECM architecture of native interfaces and avoid artifacts associated with synthetic membrane materials (e.g., non-specific drug adsorption to PDMS), but they typically sacrifice the reproducible thickness, mechanical actuation capability, and straightforward TEER integration that a defined physical membrane provides and are generally harder to standardize across replicate devices [37]. In practice, the choice is application-dependent: mechanically active interfaces requiring cyclic stretch, and platforms requiring quantitative barrier integrity readouts, generally retain a physical membrane, whereas interfaces where a 3D ECM environment and vascularization are the dominant functional requirement are increasingly implemented as membrane-free.

### 2.2. Engineering Membranes for Organ-on-a-Chip Systems

The choice of membrane for OOC systems is driven by several interconnected engineering constraints such as mechanical characterization, mass transport, surface properties, fabrication, and integration.

*Mechanical characterization.* An OOC membrane should survive suitable mechanical stimulation and fluid shear without being damaged and with a preserved membrane permeability profile. However, Young’s modulus alone is insufficient to describe this behavior: it is a material-level property measured on solid films, whereas the effective mechanical response of a thin, porous membrane also depends on the thickness, pore geometry, and clamping/bonding within the device [2]. OOC membranes not only serve as inactive supports but also as active mechanical actuators to transfer repetitive strains to cell cultures mimicking breathing movements, peristalsis, or heartbeats [5]. For stretch-based devices, the use of flexible materials, such as PDMS, is preferred because its modulus enables it to deform repeatedly without destruction. Thickness, porosity, and pore geometry should be considered jointly rather than independently, since effective bending stiffness scales with thickness while porosity reduces the load-bearing cross-section, so membranes of identical bulk modulus can show markedly different device-level compliance [56]. Native BMs are highly non-linear and strain-stiffening, while synthetic membranes cannot fully mimic that [36]. Moreover, most membrane characterization relies on single-pull tests rather than cyclic loading; PDMS itself shows a cyclic modulus that departs from its monotonic value [57], and fatigue-related changes in membrane performance over multi-week OOC culture remain largely unreported. Membrane composition tuning, such as modification of the collagen-to-elastin ratio, to achieve a mechanical range of 1 kPa to several hundred kPa, and the design of a flexible intermediate layer that homogenizes mechanical loading, are two possible solutions to this challenge.

*Mass transport.* Membrane mass transport is governed by several distinct structural. Pore size defines the physical diameter of individual openings and sets an upper limit on the size of molecules or cells that can pass. Porosity, the fraction of membrane area occupied by pores, and pore density, the number of pores per unit area, together determine the total available cross-sectional area for transport. Pore connectivity describes whether the pores form continuous channels through the membrane or isolated, impermeable voids—a parameter particularly important for fibrous or electrospun membranes where high nominal porosity does not guarantee a continuous transport path.

Membrane transport depends on much more than just thickness and porosity. Tortuosity, the ratio of the effective diffusion path length through a membrane to its straight thickness, modifies effective transport independently of pore size or porosity: two membranes with the same thickness, pore size, and porosity can differ significantly in permeability if their pore geometry is straight or tortuous. Therefore, permeability—the net rate of solute transport through an assembled membrane—is an emergent property, dependent on thickness, pore size, porosity, pore density, connectivity, and tortuosity together, and cannot be predicted from any single parameter in isolation [4,47].

Selective permeability also depends on the transported molecule itself. Molecular size relative to pore size determines whether a solute passes freely, is partially restricted (as in glomerular filtration of intermediate-sized proteins), or is excluded. Ignoring size and charge selectivity can cause non-physiological leakage or retention even when pore size and porosity appear well matched to native tissue.

*Surface properties.* Membrane surface properties (chemistry, stiffness, topology) are of equal biological importance but also largely a domain of engineering design. Both cell stiffness and topography at the nanoscale can directly affect cell differentiation and junction formation. Membrane architecture, in particular, actively shapes epithelial and endothelial polarization and junction maturation rather than merely permitting or restricting attachment: endothelial cells cultured on porous membranes form fewer focal adhesions than on continuous substrates, yet develop tight junctions earlier, indicating that pore geometry redirects cell interactions from the substrate toward neighboring cells [58]. This implies that the membrane should not be treated as a passive separator, since the same material can yield markedly different junctional maturation and barrier phenotypes depending on pore size and spacing alone. This implies that in materials design, we must account not only for the chemical but also for the mechanical features of the natural ECM. Indeed, not only does topography affect the cell, but pore topology can be engineered to determine cell morphology; smaller pores lead to cells maintaining much of their original degree of freedom in shape, while larger pores define rectangular cell morphology based on the grid [59]. Cell morphology also determined whether cells merely sampled the membrane or transmigrated fully. Membrane thickness and pore architecture further determine the mode of intercellular communication across the barrier: conventional micron-thick porous membranes primarily support paracrine signaling by diffusion through the membrane, whereas ultrathin, sub-micron membranes can bring epithelial and endothelial layers into sufficiently close, or even direct, contact to support juxtracrine processes such as podocyte foot process formation [14,59].

Surface modifications are also often unstable over time: oxygen plasma treatment of hydrophobic materials such as PDMS is prone to hydrophobic recovery within hours, typically requiring immediate cell seeding after activation [60,61]. Similarly, physically adsorbed ECM coatings lack covalent anchoring and are prone to desorption or delamination under continuous perfusion and cyclic mechanical strain [53,62], whereas covalent immobilization (e.g., polydopamine or sulfo-SANPAH crosslinking) improves adhesion but is rarely validated beyond one to two weeks of culture. Long term, quantitative data on surface modification stability under combined flow and strain remain scarce, limiting confidence in multi-week OOC applications.

*Fabrication.* The construction and incorporation of membranes into OOC systems necessitate a precise combination of micro-/nanofabrication, materials engineering, and microfluidic assembly. The main process is microfabricating the membrane and inserting it into the microfluidic system, with precise alignment and bonding tolerances. The principal element involves the actual fabrication of the membrane itself, through techniques such as micromachining, laser lithography, injection molding, spin-coating, or electrospinning, and its careful placement and bonding within a microfluidic device with stringent constraints on alignment and bonding accuracy [63].

The actual fabrication techniques are often determined by the materials used and the performance desired. Conventional polymers are best fabricated using micromolding or solvent casting, then employing either photolithography or laser micromachining to fine-tune the pore size, thickness, and uniformity. For fibrous format membranes, electrospinning becomes the technique of choice, which can produce ultrathin, high-porosity membranes with comparable ECM architectures and pore dimensions of micron to sub-micron sizes as the membrane is deposited. The ultrathin, porous nature of such membranes requires a supporting substrate during construction, so they are not damaged when transferred [64].

Electrospun membranes also present notable reproducibility challenges that are less pronounced in micromolded or lithographically defined membranes. Fiber diameter, orientation, and pore size are highly sensitive to processing parameters such as the polymer concentration, solution viscosity, applied voltage, and humidity, and small variations in these parameters between batches, or even within a single mat, can produce substantial pore heterogeneity and variability in permeability and mechanical properties [5]. Fiber orientation, whether random or aligned, further introduces anisotropic mechanical behavior and directional cell guidance cues, adding a variable that is rarely standardized across studies. In addition, pore size and fiber diameter are not independently controllable in electrospun membranes, since pores form as the interstitial space between deposited fibers: tuning the fiber diameter to achieve ECM-like nanoscale topography inherently constrains the achievable pore size range, and vice versa [65]. This coupling limits the ability to simultaneously optimize fiber-scale biomimicry and pore-scale mass transport, and represents an important, frequently unaddressed, design trade-off for electrospun OOC membranes.

*Integration.* The integration step, bonding the membrane into the microfluidic device, is often challenging. Three main integration strategies are commonly used: (i) sandwich bonding, where a pre-fabricated membrane is clamped or plasma-bonded between two PDMS or thermoplastic layers; (ii) in situ membrane generation, where the membrane is formed directly within the assembled device through gelation or biofabrication, avoiding a separate alignment step entirely; and (iii) direct membrane deposition, such as electrospinning or spin coating performed directly onto a chip substrate before bonding. Each strategy presents distinct trade-offs: sandwich bonding offers reproducibility and material flexibility but requires precise alignment to avoid dead volumes or delamination at the membrane edges; in situ generation simplifies assembly but offers less control over membrane thickness and porosity; and direct deposition can achieve excellent membrane–substrate adhesion but is constrained by the thermal and chemical compatibility of the underlying chip material. An approach to avoid having to prep the membrane simultaneously with device assembly is the use of standalone cell inserts that are prepared and cellularized in their own chamber before being inserted into the microfluidic housing, allowing simple seeding from both sides of the membrane, ensuring more uniform cell coverage, and avoiding many of the losses in cell viability and aggregation that occur when directly seeding cells into a sealed device [59]. Leakage and delamination at the membrane–chip interface present one of the challenges associated with the integration of membranes with microfluidic chips. Particularly, due to the propensity of natural and hybrid membranes to swell, the chips integrated with these may often result in faster delamination at the bonded layer interface under perfusion and cyclic mechanical strain [54].

The long-term practicality of OOC devices requires the membrane–chip interface to tolerate many use cycles and fluidic exchanges while maintaining consistent performance during sterilization procedures, thus making the integration scheme itself as important as the selection of the membrane material.

## 3. Types of Membrane-Integrated Organ-on-a-Chip Platforms

Membrane-integrated organ-on-a-chip (OOC) platforms can be categorized based on the physiological barrier they mimic. The membrane serves as a critical interface that separates microchannels while supporting cell adhesion, nutrient transport, and mechanical cues. Table 1 shows the main membrane parameters and their functions in different OOC systems with representative example studies. Importantly, while membrane-integrated OOC platforms are frequently categorized by target organ, many underlying membrane design principles—such as permeability control, mechanical compliance, and surface biofunctionalization—are shared across systems. Comparative analysis of different organ models therefore provides valuable insight into common design constraints and transferable membrane engineering strategies.

*Alveoli–capillary interface-on-a-chip.* Alveoli–capillary interface-on-a-chip platforms [7,8,9,10,12,66] use biomimetic membranes to recreate the alveolar–capillary barrier, and recent reviews highlight that the membrane structure (e.g., pore size, composition, mechanical compliance) is critical for mimicking physiological lung interfaces [37]. Figure 2 illustrates representative lung-on-a-chip (LOC) microsystems with different membrane types.

The natural alveoli–capillary membrane in the human lungs has a 0.1 to 2 µm thickness and is mainly composed of collagen IV, laminin, and proteoglycans [70]. This basement membrane (BM) has a fibrous topology with the concomitant presence of pores ranging between 10 and 150 nm. When it comes to mechanical properties, an alveolar–capillary membrane is reported to have a Young’s modulus in the range of 2 MPa [71].

Hydrogel-based stretchable membranes have been developed to replace conventional polydimethylsiloxane (PDMS) films, finding that a soft, extracellular matrix (ECM)-mimicking membrane improved epithelial–endothelial barrier formation and reduced fibrotic responses under cyclic strain [6]. This shift toward softer and more elastic membranes reflects an increasing emphasis on reproducing physiologically relevant mechanical cues rather than solely focusing on device manufacturability. Additionally, nanofibrous membranes fabricated via electrospinning to mimic the lung BM have been incorporated into alveolar interstitium chips, enabling extended culture times and improved barrier function [8]. Hybrid membrane approaches have also become available; a polycaprolactone (PCL)–collagen nanofiber hybrid membrane (with a thickness of about 10 µm) has been used in LOC microfluidic models, which enables scaffolding BM structures more efficiently and allows them to be more biocompatible [66]. Such ECM-mimicking architectures further highlight the role of the membrane microstructure in regulating epithelial organization and barrier maturation. These are competing, not complementary, solutions: PDMS [9] offers predictable, reproducible mechanics but a stiffness far from native BM values; hydrogel membranes [6] better match native stiffness but degrade faster under perfusion; and hybrid nanofiber membranes [8,66] sit in between, trading fabrication simplicity for improved longevity and fidelity.

*Glomerular filtration barrier-on-a-chip.* Glomerular filtration barrier-on-a-chip platforms [16,18,19] involve an artificial renal barrier system based on semipermeable membranes that mimic the functions of filtration, secretion, and reabsorption in the kidney. Different membrane-based kidney and glomerulus-on-a-chip models are illustrated in Figure 3. These membranes serve as physical and biochemical interfaces separating microchannels that represent the vascular and tubular compartments, but they permit the movement of molecules and cell–cell signaling [38]. A human kidney chip consisting of an ultrathin polymer membrane that allowed direct contact between the endothelial and epithelial layers was designed, which is a feature of the biological setting of selective permeability and formation of podocyte feet [14]. Similarly, a glomerulus-on-a-chip was fabricated with a tunable BM layer that supported three-dimensional podocyte cultures and recapitulated native filtration behavior under dynamic flow [15]. These findings illustrate that membrane thickness and permeability must be tightly controlled to maintain selective filtration while preventing non-physiological leakage. The results highlighted here are that membrane morphology and compliance play a pivotal role in the preservation of the renal barrier in microfluidic conditions. The polarized proximal tubule epithelial cells require this to be maintained through the membrane in tubular kidney-on-a-chip (KOC) systems that can withstand physiological shear stresses and osmotic gradients. Importantly, membrane performance in these systems is closely linked to its ability to withstand physiological shear stress without compromising epithelial polarization. Contrast-induced kidney injury using a three-dimensional KOC based on a porous polymer membrane was assessed, in which both kidney membrane osmolality and viscosity had a direct impact on cell viability and reabsorption [19]. Ultrathin membranes [14] better replicate native filtration selectivity but are mechanically weaker under shear/osmotic stress than thicker, porous membranes [18,19], which trade selectivity for durability—no reported design achieves both simultaneously. The deposition of ECM-like materials on synthetic membranes improves epithelial–endothelial co-culture integration and barrier tightness [15]. Moreover, recent reviews report a growing shift toward ECM-based or hydrogel-derived membranes, which more accurately reproduce native BM stiffness and permeability, reducing non-physiological artifacts observed with traditional PDMS substrates [38]. Taken together, this shows that the fine-tuning of membrane characteristics, including thickness down to the sub-micron scale, porosity that is neither too permeable nor too selective, and elasticity that allows cyclic flow, is also necessary to make KOC systems more physiological.

*Hepatic sinusoidal interface-on-a-chip.* Hepatic sinusoidal interface-on-a-chip systems [22] have emerged as microphysiological systems that are intended to mimic the structure and functionality of the human liver in vitro. Liver-on-a-chip within the framework of biomimetic membranes consists of replicating the hepatic sinusoidal interface, which supports nutrient and oxygen circulation as well as co-cultures of hepatocytes and endothelial cells [72]. Incorporation of porous membranes coated to mimic the BM in the space of Disse, such as gelatin-coated PC/PET membranes, has been shown to increase hepatocyte viability and albumin secretion under perfused conditions [20]. Mimicking this highly specialized interface places stringent requirements on membrane porosity and surface chemistry. The reviews of these microfluidic platforms also note that the choice of membrane material, pore size, and surface modification is a critical design parameter in enhancing the physiological fidelity and translation potential of such microphysiological systems [73]. Recent developments in membrane surface modification—such as plasma treatment or ECM patterning—further promote cell attachment and enhance hepatocyte–endothelial communication in dynamic perfusion systems [72]. These improvements underscore how subtle modifications in membrane design can significantly impact long-term hepatic function under perfused conditions. Large-scale validation work has also shown that a well-engineered membrane-enabled liver chip can outperform conventional in vitro models for predicting drug-induced liver injury, underscoring the translational importance of membrane design [21]. In general, all these developments underscore the critical necessity of membrane engineering to design physiologically reliable liver chip systems that could be used in testing drugs and modeling diseases.

*Neurovascular barrier-on-a-chip.* Many membrane-integrated blood–brain barrier-on-a-chip (BOC) models are built using a porous polymer membrane to isolate a vascular channel and a parenchymal chamber and, therefore, recreate the BOC luminal–abluminal architecture. Multiplexed BOC was used in a study and presented a two-layer device made of PDMS with a transparent 5 µm thick membrane [74]. A membrane-based microfluidic BBB model was used to test the permeability of 10 compounds, which showed that a membrane-integrated BOC could be used to screen drugs [25]. Scientists further enhanced the platform by integrating transepithelial electrical resistance (TEER) electrodes adjacent to the membrane, enabling real-time monitoring of the integrity of the barriers and the transport of nanoparticles [24]. This indicated that porous membranes were crucial in forming tight junctions as well as the selective transport of molecules. Such precise control over membrane architecture is essential for capturing the extreme selectivity of the BBB. Another study indicated that ultrathin silicon nitride membranes would become a good scaffold to co-culture endothelial cells and astrocytes [27]. This provided better optical transparency for imaging and very good control of permeability measurements. At the same time, reducing the membrane thickness introduces trade-offs between optical accessibility, mechanical robustness, and fabrication yield. In addition, the disruption of barriers, inflammation, and T-cell migration of the endothelium is exposed by a human BOC in neuroinflammatory conditions [26]. All these works confirm the point of view according to which the application of porous membranes is the key to the physiologically relevant functions of human barriers.

*Intestinal epithelial barrier-on-a-chip.* Intestinal epithelial barrier-on-a-chip systems [32] have been developing swiftly to duplicate the complex design and dynamic setting of the human intestinal epithelium. Fluid flow and mechanical stimulation are usually used in these devices to recreate peristaltic motions, which greatly improve epithelial differentiation, microvilli formation, and barrier integrity over standard culture systems [29]. These dynamic conditions place additional mechanical demands on membranes compared to static intestinal models. The interactions between the host and microbiome under dynamic conditions can be studied with the help of co-culture strategies in such chips, such as human intestinal epithelial cells and commensal microbes [75]. Consequently, membrane permeability must be carefully balanced to allow controlled microbial interaction while preserving epithelial barrier integrity. Electrode-integrated membrane designs have also been reported for real-time monitoring of intestinal barrier integrity, where electrodes were integrated directly onto the membrane to enable spatially resolved TEER measurements [76]. Next-generation ex vivo dual-flow intestinal mucosal barrier models also allow the study to be more precisely controlled using disruption in the mucosal barrier (e.g., dose-dependent breakdown of collagen-I and claudin-1 by bacterial enzymes) into a leaky gut model of disease research [30]. Moreover, these devices are used to support translational use, such as the evaluation of drug carrier transit and microbial translocation across the intestinal barrier [31]. Finally, the microenvironment complexity of these systems, such as shear stress, perfusion, and microbial co-culture, has made the platform a very good platform to study intestinal physiology, disease pathophysiology, and therapeutic approaches.

## 4. Future Perspectives

Despite the great advancement in the field of organ-on-a-chip (OOC) technology, particularly in membrane-integrated platforms and the accelerating growth of different types of OOC devices, the development of suitable biomimetic porous membranes for OOC systems remains slow [47]. To better mimic the native ultrathin basement membrane, membrane thickness remains one of the existing challenges. While thinner membranes allow for higher hydraulic and diffusive permeability, they often do not have in vivo-like membrane surface and stiffness properties. Artificial membranes commonly used in in vitro models and OOCs do not replicate the characteristics of natural basement membranes (BMs) [55]. Many of the reported membranes used in existing OOCs have stiffer mechanical properties than a native membrane. This mismatch is not resolved by switching membrane class alone—synthetic membranes overshoot native stiffness, natural membranes undershoot durability, and hybrids only partly close both gaps. Head-to-head comparisons under identical conditions are needed to move membrane selection onto quantitative grounds. Long-term stability also remains a challenge, as natural and hybrid membranes are prone to degradation, swelling, or delamination under continuous perfusion and cyclic mechanical strain, limiting their suitability for chronic disease models and repeat-dose drug testing [54].

Additionally, since the main aim of OOC technology is drug testing, OOC platforms need to have consistency and accuracy in serial production. Therefore, the reproducibility of such ultrathin membranes also needs to be improved not only in their standalone form but also in their integrated form within microfluidic platforms. The requirement for specialized skills for consistent and reproducible microfabrication currently limits the broader adoption of OOC devices outside of the research community [77]. Currently, the majority of the reported OOC devices integrated with membranes present proof-of-concept work with limited suitability for mass production or high-throughput studies. Advancement of hybrid and “smart” membrane types that possess biomimetic content, mechanical, and surface properties needs to proceed and be perfected before these membranes can be integrated within microfluidic devices for more robust, reproducible, and efficient OOC platforms. In practice, “smart” behavior means that membranes are designed to change porosity, stiffness, or permeability in response to a specific stimulus—for example, pH, temperature, mechanical stress, or an applied electric field. This allows the membrane to actively reproduce dynamic physiological changes rather than serve as a static barrier. The development of materials that respond to external stimuli and allow control of material properties over time adds a level of complexity that may provide fundamental insights into cellular behavior [77]. The convergence of biomaterials science with microfluidics—including the integration of surface coating components, protein-based membranes, and hydrogel-based constructs—is seen as a promising step toward a more realistic reconstruction of the tissue-level three-dimensional microarchitecture within OOC systems [78].

Development of biomimetic membranes is key in the field of membrane-integrated organ-on-a-chip. Membrane-integrated OOC systems bridge traditional in vitro cell culture and in vivo organ physiology, with membranes acting not only as structural separators but also as dynamic interfaces regulating mass transport, mechanical cues, and cell–cell communication [5,9]. The evolution of membrane materials, from polydimethylsiloxane (PDMS) and conventional polymers to biologically derived hydrogels and synthetic/hybrid composites, reflects the critical need to balance mechanical robustness, biocompatibility, and manufacturability [2,5]. On the fabrication side, advances in micromolding, electrospinning, laser lithography and additive manufacturing, together with refined bonding and integration strategies, are enabling more precise control of membrane geometry, porosity, thickness and surface chemistry, allowing the membrane to act both as a passive barrier and an active sensing/actuation element [5,47]. Organ-specific implementations—from lung to kidney, liver, gut, and blood–brain barrier models—illustrate how tailored membrane architecture and material choice make physiological relevance and translational application possible [71]. Moving forward, the key to the broad adoption of OOC platforms lies in standardizing the membrane materials and integration workflows, ensuring scalable manufacturability, and embedding multifunctionality (sensing, actuation, interconnectivity) at the membrane–chip interface so that these systems not only mimic tissue physiology but are robust, reproducible, and ready for industry-scale deployment.

## Figures and Tables

**Figure 1:**
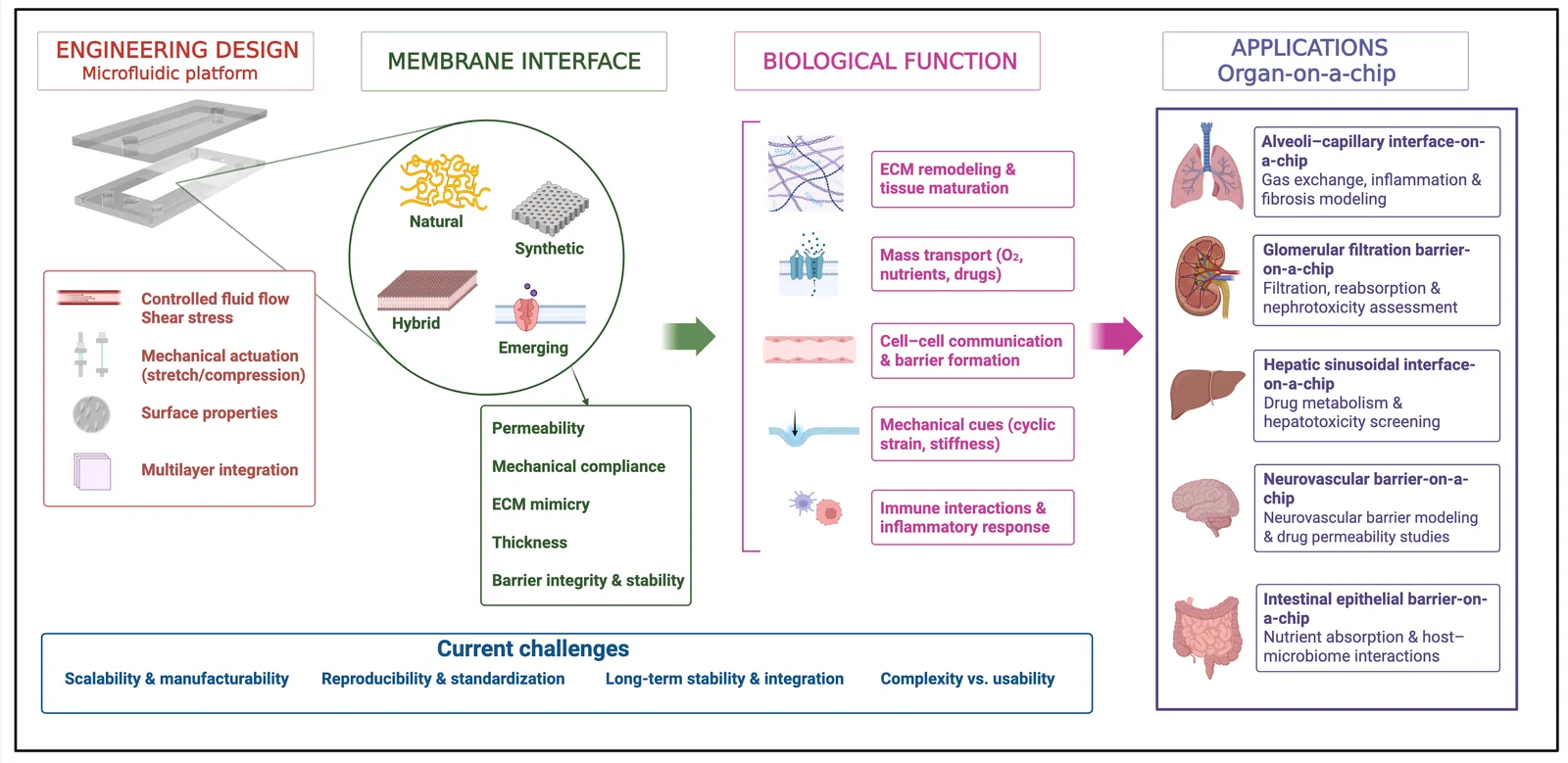
Schematic representation of membrane-integrated microfluidic platforms for organ-on-a-chip applications: from design to functions. Created in https://BioRender.com.

**Figure 2:**
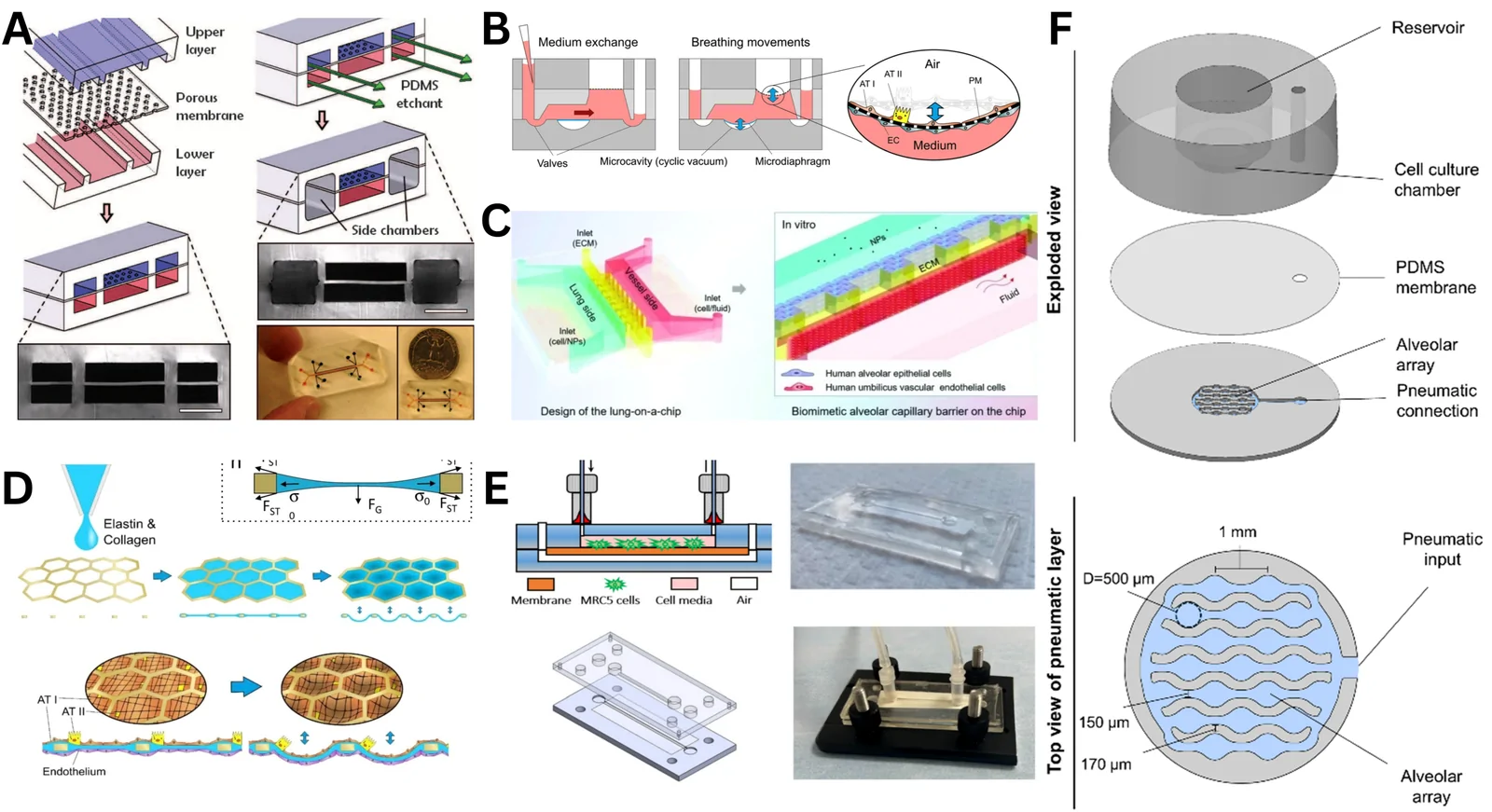
Different studies conducted using different LOC designs. (**A**) PDMS membrane with through-holes and side vacuum chambers. Adapted from Huh et al. [9], AAAS. (**B**) Schematic cross-sections of the LOC with two operation modes. Adapted from [67], licensed under the Creative Commons Attribution 4.0 International License (CC BY 4.0). (**C**) LOC design and alveolar–capillary barrier [68]. Reproduced under the Royal Society of Chemistry license. (**D**) Collagen–elastin membrane and alveolar cell co-culture. Adapted from [12], licensed under the Creative Commons Attribution 4.0 International License (CC BY 4.0). (**E**) Nanofiber membrane-integrated lung-on-a-chip design and assembly. Adapted from Kanabekova et al. [66]; permission conveyed through Copyright Clearance Center, Inc., on behalf of the Royal Society of Chemistry. (**F**) Three-layer LOC with pneumatically controlled alveolar array. Adapted from [69], licensed under the Creative Commons Attribution 3.0 License (CC BY 3.0).

**Figure 3:**
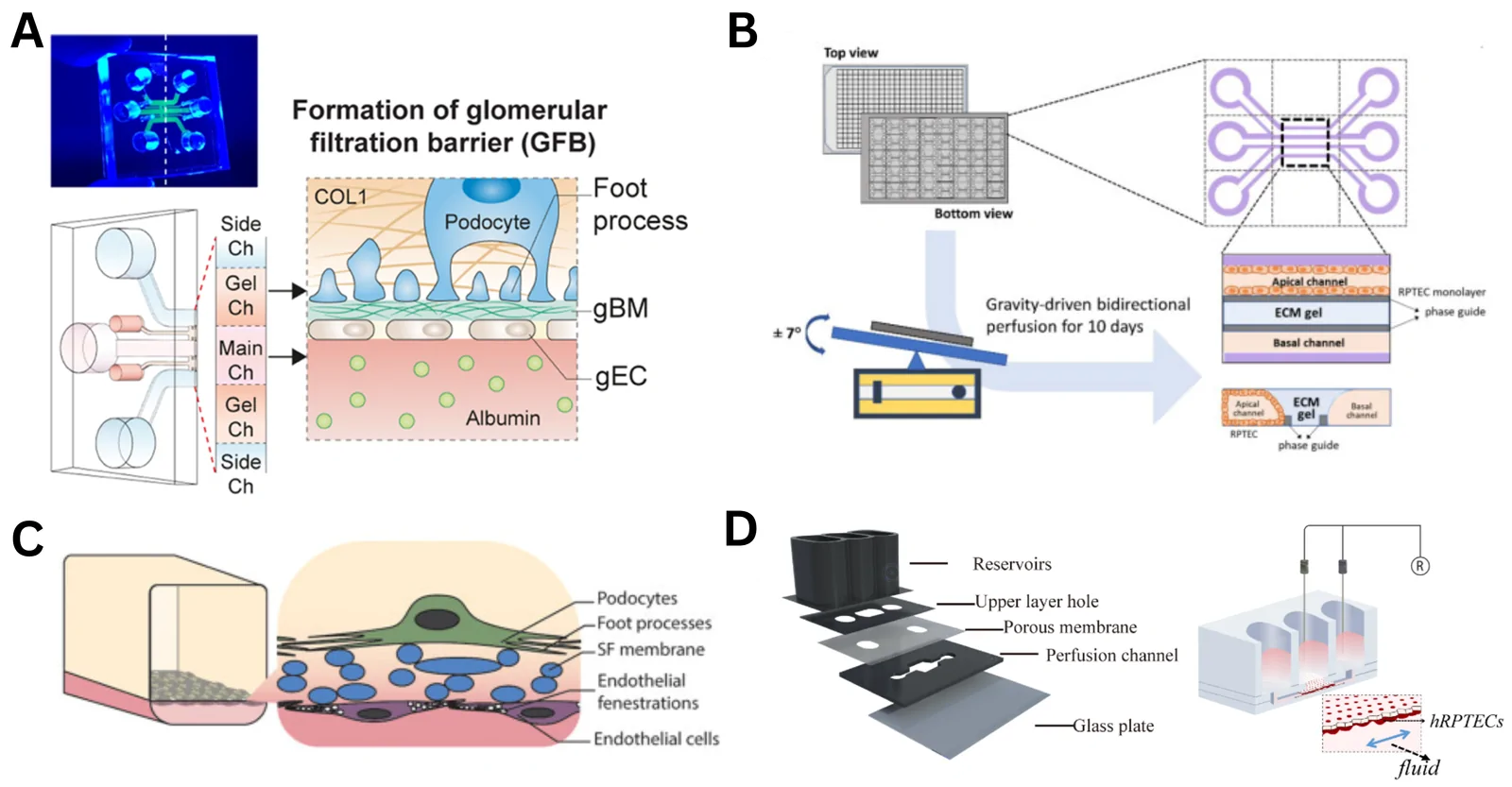
Diversity of membrane strategies in kidney and glomerulus-on-a-chip systems. (**A**) Three-dimensional glomerular filtration barrier-on-a-chip model. Adapted from Kim et al. [15]; permission conveyed through Copyright Clearance Center, Inc., on behalf of the Royal Society of Chemistry. (**B**) 3D tubular structure in the three-lane OrganoPlate. Adapted from [19] under the Creative Commons Attribution 4.0 International License (CC BY 4.0). (**C**) Engineered tissue model cross-sectional structure. Adapted from Mou et al. [14], AAAS. (**D**) Integrated biomimetic array chip for renal tubule cell culture. Adapted from [18], licensed under the Creative Commons Attribution 4.0 International License (CC BY 4.0).

**Table 1:** Membrane design characteristics across membrane-integrated organ-on-a-chip platforms.

OOC Model	Membrane Material	Thickness Range (µm)	Native BM Thickness	Mechanical Requirements	Key Disease/Application Model	Main Functional Outcomes	Ref.
Alveoli–capillary interface-on-a-chip	PDMS, hydrogel membranes, electrospun nanofibers, porous silicon	~5–15	~0.2 µm	Cyclic stretch (5–10%), elastic compliance	Radiation-induced lung injury, nanotoxicology, pulmonary fibrosis	Barrier integrity, inflammatory response, gas exchange	[6,7,8,9,10,11]
Alveoli–capillary interface-on-a-chip (hybrid)	PCL-collagen, collagen–elastin nanofibers	4.5–11.5	~0.2 µm	Enhanced biocompatibility	Basement membrane mimicry for lung disease modeling	Improved epithelial–endothelial interaction	[11,12,13]
Glomerular filtration barrier-on-a-chip	Ultrathin polymer (~50× thicker than native GBM), ECM-coated membranes	<1–2	0.29–0.32 µm	High compliance under flow	Diabetic nephropathy, proteinuria; drug-induced podocyte injury	Albumin retention, podocyte–endothelial interaction	[14,15,16,17]
Proximal tubule-on-a-chip	Porous polymer membranes	5–20	0.29–0.32 µm	Resistance to shear stress	Nephrotoxicity testing, cisplatin-/contrast-induced AKI	Transporter function, tubular reabsorption	[17,18,19]
Hepatic sinusoidal interface-on-a-chip	PC/PET membranes with ECM or gelatin coating	10–30	~0.2–1 µm	Stable perfusion, low shear	Drug-induced liver injury, hepatotoxicity screening	Albumin secretion, CYP activity, hepatotoxicity screening	[20,21,22,23]
Neurovascular barrier-on-a-chip	Porous polymer membranes, silicon nitride	~1–2	~0.2 µm	Structural stability, tight junction support	Neuroinflammation; Alzheimer’s disease, MS drug testing	TEER monitoring, drug permeability	[24,25,26,27,28]
Intestinal epithelial barrier-on-a-chip	Flexible porous membranes (collagen-coated polymers)	10–50	3–20 µm	Cyclic strain (peristalsis-like motion)	IBD, gut permeability, host–pathogen interaction	Microvilli formation, host–microbiome interaction	[29,30,31,32,33]

Abbreviations: OOC, organ-on-a-chip; PDMS, polydimethylsiloxane; PCL, polycaprolactone; ECM, extracellular matrix; PC, polycarbonate; PET, polyethylene terephthalate; CYP, cytochrome P450; AKI, acute kidney injury; TEER, transepithelial electrical resistance; MS, multiple sclerosis; GBM, glomerular basement membrane.

## Data Availability

No new data were created or analyzed in this study. Data sharing is not applicable to this article.

## Abbreviations

The following abbreviations are used in this manuscript:

OOC	organ-on-a-chip
BM	Basement membrane
LOC	Lung-on-a-chip
PDMS	Polydimethylsiloxane
COC	Cyclic olefin copolymer
PCL	Polycaprolactone
ECM	Extracellular matrix
KOC	Kidney-on-a-chip
TEER	Transepithelial electrical resistance
BBB	Blood–brain barrier
BOC	Blood–brain barrier-on-a-chip
